# Surgery Combined with Local Implantation of Doxorubicin-Functionalized Hydroxyapatite Halts Tumor Growth and Prevents Bone Destruction in an Aggressive Osteosarcoma

**DOI:** 10.3390/jfb15080232

**Published:** 2024-08-19

**Authors:** Yang Liu, Tova Corbascio, Jintian Huang, Jacob Engellau, Lars Lidgren, Magnus Tägil, Deepak Bushan Raina

**Affiliations:** Department of Clinical Sciences Lund, Orthopedics, The Faculty of Medicine, Lund University, 221 00 Lund, Sweden; yang.liu@zju.edu.cn (Y.L.); tova.corbascio.5146@student.lu.se (T.C.); jintian.huang@med.lu.se (J.H.); jacob.engellau@med.lu.se (J.E.); lars.lidgren@med.lu.se (L.L.); magnus.tagil@med.lu.se (M.T.)

**Keywords:** osteosarcoma, drug delivery, hydroxyapatite, doxorubicin, animal model

## Abstract

Osteosarcoma treatment comprises pre-surgical chemotherapy followed by radical surgery and further chemotherapy cycles, but the prognosis has been far from satisfactory. No new drugs or treatment modalities have been developed for clinical use in the last four decades. We describe a nano-hydroxyapatite (HA)-based local drug delivery platform for the delivery of doxorubicin (DOX), a cornerstone drug in osteosarcoma treatment. The efficacy of the developed drug delivery system was evaluated in an orthotopic human osteosarcoma xenograft in the proximal tibia of mice. After tumor development, the tumor was surgically resected and the void filled with the following: (1) No treatment (G1); (2) nHA only (G2); (3) DOX-loaded nHA (G3). In-vivo tumor response was assessed by evaluating the tumor-induced osteolysis at 2 weeks using micro-CT followed by in-vivo PET-CT at 3 weeks and ex-vivo micro-CT and histology. Micro-CT imaging revealed complete destruction of the tibial metaphysis in groups G1 and G2, while the metaphysis was protected from osteolysis in G3. PET-CT imaging using ^18^F-FDG revealed high metabolic activity in the tumors in G1 and G2, which was significantly reduced in G3. Using histology, we were able to verify that local DOX delivery reduced the bone destruction and the tumor burden compared with G1 and G2. No off-target toxicity in the vital organs could be observed in any of the treatment groups histologically. This study describes a novel local drug adjuvant delivery approach that could potentially improve the prognosis for patients responding poorly to the current osteosarcoma treatment.

## 1. Introduction

Recent decades have witnessed novel cancer therapies, including CAR-T treatment, immunotherapy and theranostic-based approaches [1]. However, when it comes to solid tumors of the bone, and in particular, osteosarcoma, no new treatment approach or chemotherapeutic agent has been introduced in the past four decades. The MAP (methotrexate, adriamycin and cisplatin) protocol is still the standard treatment regimen for patients diagnosed with osteosarcoma and the combination is administered for two cycles before a radical surgical resection of the tumor is performed. During the post-surgery period, MAP treatment is continued for four more cycles, according to the Euramos-1 study [2]. Malignant tumors of the bone have been treated by surgical resection since the 19th century [3]. The goal of the surgery is to remove the entire tumor or as much of it as possible. Wide resection of the tumor, including a margin of assumed healthy tissue, is a standard surgical procedure. However, in some cases complete resection of the tumor is impossible and there is always a risk of residual cells, especially in high-grade malignant tumors [4]. In osteosarcoma, 15–25% patients have a relapse (local recurrence or metastases) after surgical resection even with wide ablation [5]. Furthermore, it has been reported that recurrent osteosarcoma occurs in 30–50% of patients with an initial localized disease and 80% of patients presenting with a metastatic disease [6,7]. 

In general, chemotherapy, introduced in the 1970s, has played a vital role in killing the remaining cancer cells close to the resected area as well as inducing cell death in micro-metastasis. This systemic effect achieved by chemotherapy has in many cancer types improved the prognosis and the long-term survival of the patient [8]. For osteosarcoma, the standard MAP protocol or an extended MAPIE (MAP+ ifosfamide and etoposide) has been used for more than 40 years without any new drugs being invented [9,10]. We know there is a large number (>30–40%) of patients responding poorly to the current cytostatics, and the 5-year survival rate is low (50%) [7,11]. Furthermore, these cytostatics are usually administered intravenously or sometimes intraarterially. After being injected via the circulation, most cytostatics accumulate in vital organs like the liver, kidney and lungs, causing severe side effects. Only 5–10% of the systemically administered drugs reach the local tumor site [12], leading to poor treatment response. Under these conditions, the remaining cancer cells may regrow, both locally and at far sites, and therefore may induce recurrence and further metastasis.

Compared to systemic administration, local drug delivery is a promising and attractive way to release a large fraction of drug molecules at or near the site of action and thereby reduce off-target side effects [13,14,15]. Furthermore, the delivery system allows a sustained drug release, which impedes local destruction and therefore also alleviates the negative impact on the healthy surrounding area [16]. We have previously shown that doxorubicin (DOX), one of the key drugs in the MAP regimen, interacts electrostatically with synthetic hydroxyapatite (HA) crystals. HA is the major inorganic component of our bones and comprises nearly 65 wt% of our skeleton. In a recent study, we demonstrated a sustained and controlled release of doxorubicin (DOX) from calcium sulfate/micro-sized hydroxyapatite (CaS/HA) [17]. As a continuation of this study, we explored the role HA particle size plays in DOX delivery and found that by using nanoparticulate HA (nHA), intracellular DOX delivery could be achieved. Intracellular DOX delivery via the HA particles targeted the mitochondria of the human osteosarcoma cell line 143B [18]. After testing the in vitro effect of the nHA-DOX combination, we tested the developed drug delivery system on an in-vivo subcutaneous osteosarcoma model using 143B cells in nude mice, and could demonstrate that the local and controlled DOX delivery had a significantly better effect on halting the tumor growth than administering DOX systemically. The 143B human osteosarcoma cell line is one of the most aggressive cancer cell lines used in the study of osteosarcoma and shows an osteolytic phenotype inducing bone damage [19]. In our previous studies, we have not investigated the efficacy of nano-HA-mediated DOX delivery on a clinically relevant orthotopic osteosarcoma in mice. Therefore, the aim of this study was to evaluate the efficacy of local DOX delivery using HA nanoparticles in an aggressive orthotopic xenograft in nude mice, which better mimics the osteosarcoma tumor microenvironment, including in tumor progression and bone osteolysis.

## 2. Materials and Methods

### 2.1. Materials Preparation

Doxorubicin hydrochloride was purchased from Merck (Berlin, Germany) and nano-hydroxyapatite paste was procured from Fluidnova (Maia, Portugal). Cell culture reagents including RPMI medium and Glutamine were both purchased from Thermo Scientific (Lenexa, KS, USA). Fetal bovine serum (FBS) for cell culture as well as cell viability reagent (MTT) were both purchased from Sigma Aldrich (Hamburg, Germany). Human osteosarcoma cell line 143B was bought from ATCC (Manassas, VA, USA). Experimental mice (Athymic nude Fox1^nu/nu^) were bought from Janvier Labs (Bonchamp-lès-Laval, France). 

### 2.2. Study Design and the Development of an Orthotopic Osteosarcoma Xenograft Model

**Figure 1:**
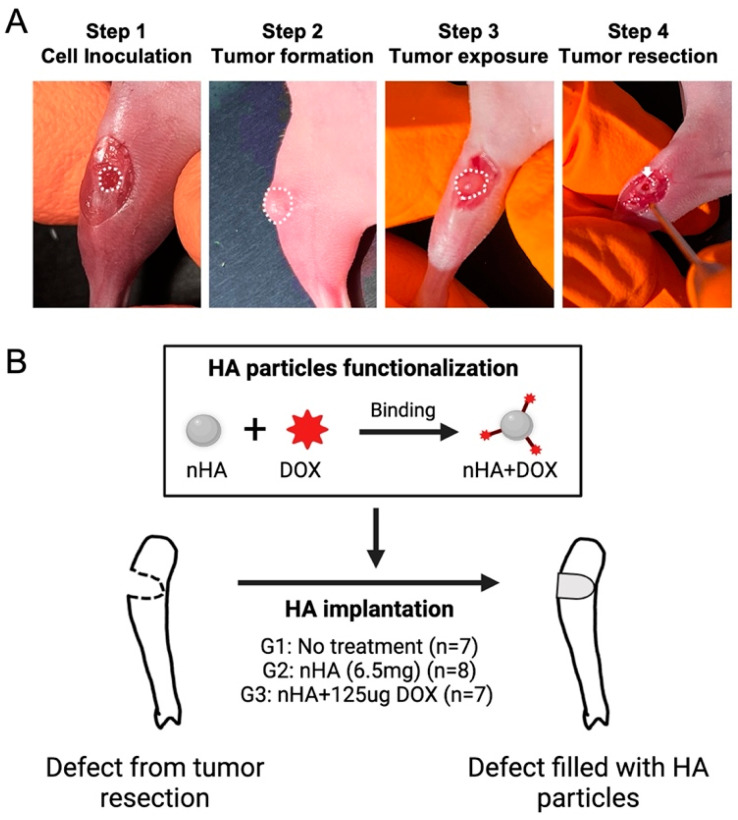
Orthotopic xenograft construction, tumor resection and HA particles implantation. (**A**) shows the procedures used for establishing the orthotopic xenograft and tumor resection after a matured tumor was established in the bone. The white dashed line indicates the cell inoculation site and matured tumor. The white arrow indicates the defect created after tumor resection. (**B**) schematic shows the design of this study: HA nanoparticles were first functionalized with DOX and then implanted in the cavity formed after the tumor resection. This figure was partly made on BioRender.com.

The 143B human osteosarcoma cells were first 2D cultured in T75 culture flasks with RPMI 1640 medium, supplemented with 10% fetal bovine serum and 1% penicillin-streptocillin mixture. Then, 2D cell culture was performed until the cells reached a confluence of approximately 80%, following which the monolayer was harvested using trypsin-EDTA solution followed by cell counting. In total, 2 × 10^5^ 143B human osteosarcoma cells were seeded on a collagen scaffold (5 mm × 2 mm) and cultured for 48 h in order to ensure accurate placement and retention of the cells locally during the animal study. On the day of surgery, the mice were anesthetized using isoflurane anesthesia using well established protocols after which the proximal tibia was exposed. A hole (Ø = 2 mm) was made by a handheld drill in the proximal tibia of the mice and was filled with the collagen scaffold containing the 143B cells. Ten days after cell inoculation, the tumor tissue was debrided using a 2 mm drill and the tumor margins were left intact in order to mimic the post-resection clinical scenario (Figure 1A). Finally, the drilled hole was filled with DOX-functionalized nHA particles. The nHA particles used in this study have been physiochemically characterized in a previous study published by our group. The particles have a rod-like shape with a majority of particles ranging between 20–50 nm, with a phase purity of >99% as observed with X-ray diffraction [18]. In total, 22 mice were randomly divided into 3 groups: 1. No treatment (G1, *n* = 7); 2. Pristine nHA (G2, *n* = 8); 3. nHA + DOX (G3, *n* = 7) (Figure 1B). For G2 and G3, 6.5 mg nHA particles was filled into the debrided void. For G3, 6.5 mg nHA particles containing 125 µg DOX per animal was used. nHA particles were functionalized using a previously developed method [18]. Briefly, 45 mg nHA particles was added to a 2 mL tube containing 1 mg DOX solubilized in 1000 µL saline. The nHA particles were mixed thoroughly with DOX using a vortex mixer for 48 h. After the mixing, the tube was centrifuged at 5000 rpm for 2 min to allow for all HA particles to settle down. At this time, the supernatant was removed from the tube and the HA particles were washed with 1000 µL saline 2x, and each time the saline supernatant was collected. The amount of DOX in the first supernatant as well as in the washed fractions was then measured using a spectrofluorimeter at an excitation of 485 nm and an emission of 580 nm in order to determine the total bound DOX. Using this functionalization approach, 6.5 mg of nHA particles contained 125 µg DOX, amounting to a loading efficacy of approximately 88%. The dose of DOX in this study is based on the maximum tolerated dose (6 mg/kg) of systemically administered DOX in mice [20]. All animals were sacrificed 21 days post treatment initiation with nHA + DOX. 

### 2.3. Detection of Metabolically Active Tumor Volume Using PET-CT

A Nano PET/CT scanner (Mediso, Hungary) was used to measure the metabolically active tumor volume 20 days post-intervention, using ^18^F-Fluorodexoyglucose (^18^F-FDG). ^18^F-FDG (43.4 ± 5.1 MBq) saline was injected intraperitoneally in a 100–500 μL final volume in awake mice, following which the mice were kept warm at 30–35 °C for a period of 45 min. The mice were maintained at a higher temperature to reduce the non-specific uptake of the tracer in fat tissues. Forty-five minutes after the tracer injection, a CT scan of the animal was acquired in order to assign skeletal landmarks that would later help in evaluating the origin of the PET signal. Finally, at *t* = 62 ± 2 min, PET imaging was performed at a resolution of 300 μm using a small animal PET/CT scanner and the images were 3D-reconstructed using manufacturer’s software suite, as described previously [17]. Regions of interests were drawn over the tumor area semi-automatically in order to quantify total ^18^F-FDG consumption in each tumor, as we have described in an earlier study [17].

### 2.4. Tumor-Induced Bone Destruction Evaluated by Micro-CT

Two weeks after treatment, all animals were imaged using an in-vivo micro-CT (MiLabs U-CT) scanner under isoflurane anesthesia. CT images were post-reconstructed to a voxel size of 30 µm and 3D models of bones were created using Seg3D2 (University of Utah, Salt Lake City, UT, USA) in order to evaluate the extent of bone destruction at the tumor site. Same micro-CT procedure was repeated at the end of the experiment on day 21. However, the imaging at the terminal time point was performed ex vivo using the harvested bones, using the same instrument, and the images were reconstructed to 20 µm voxel size. Following scan settings were used during the micro-CT investigations: magnification-ultra-focus, scan angle-360 degrees, angle step degree-0.25, binning-1:1, exposure time-75 ms, X-ray voltage-50 kV and X-ray current-0.21 mA. 

### 2.5. Hematoxylin and Eosin Staining of the Tumor-Bearing Tibia

Histology was used to compare the microstructure of the harvested tumor tissues as well as to evaluate the extent of bone destruction in each treatment group. Samples from each group were fixed in 4% (*v*/*v*) formalin solution for 24 h and decalcified in 10% *w*/*v* EDTA (pH 7.4, 4 °C) for 4 weeks with fresh EDTA changes twice every week. Routine sample dehydration using ethanol followed by xylene was performed, following which the paraffin-embedded blocks were cut to a thickness of 5 µm using a semi-automatic microtome (HM355S, Thermo Fisher Scientific, MA, USA). All samples (*n* = 22) were stained with hematoxylin and eosin (H&E) staining and digitized on a Hamamatsu slide scanner. Four samples (1 in G1, 2 in G2 and 1 in G3) had to be excluded from the quantification of bone area due to technical difficulties that arose during sectioning. The included samples were rearranged in ImageJ with the knee joint on the upper side. Then the bone area (mm^2^) in the proximal tibia was quantified using the same region of interest on all image sets. 

### 2.6. Animal Ethics Statement

All animal procedures were approved by the Swedish Board of Agriculture (Ethical approval number 5.8.18-01018/2020) and performed in accordance with the directives of the Swedish regulatory authority for the use of experimental animals. Furthermore, we have also adhered to the ARRIVE guidelines for providing information pertaining to the animal experimentation.

### 2.7. Statistical Analysis

Data are presented as mean ± SD and checked for normality (Shapiro–Wilk test) before statistical analysis. In order to statistically compare the outcome of tumor weight analysis as well as the analysis of ^18^F-FDG uptake in the tumor, a parametric ANOVA with Tukey’s multiple comparison was performed. *p*-value <  0.05 was set to be statistically significant. All data processing was carried out in Prism8, v8.2.1 (GraphPad, San Diego, CA, USA).

## 3. Results

### 3.1. Tumor Progression after Surgical Resection Followed by Local Implantation of DOX-Functionalized HA Nanoparticles

**Figure 2:**
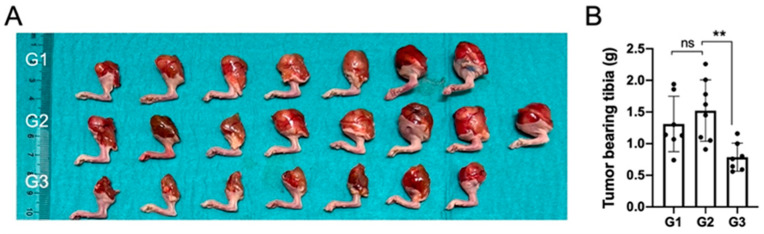
Local DOX delivery via an nHA-based carrier halts growth of an aggressive orthotopic osteosarcoma. (**A**) shows a digital photograph of all the tumor-bearing tibiae collected after sacrifice at 3 weeks. (**B**) shows weight of all the collected tumor-bearing tibia. ** indicates *p* < 0.01, ns = non-significant.

In terms of gross macroscopic pathology, the proximal tibiae in groups G1 and G2 had visible and palpable tumors on all specimens and the size of the proximal tibia was larger when compared to nHA + DOX-treated G3 specimens (Figure 2A).

The proximal tibia in G3 specimens was smaller than the proximal tibia in the specimens in G1 and G2, and four out of seven specimens appeared normal macroscopically. These findings were reflected in the net weight of the harvested tibiae and the tibia in G3 weighed significantly lower than in G2, which was treated with pristine nHA, indicating that the addition of DOX to the nHA particles was important to reduce the tumor size (Figure 2B). 

### 3.2. Surgical Resection Followed by Local Implantation of nHA + DOX Composites Drastically Reduce Tumor Metabolic Activity

**Figure 3:**
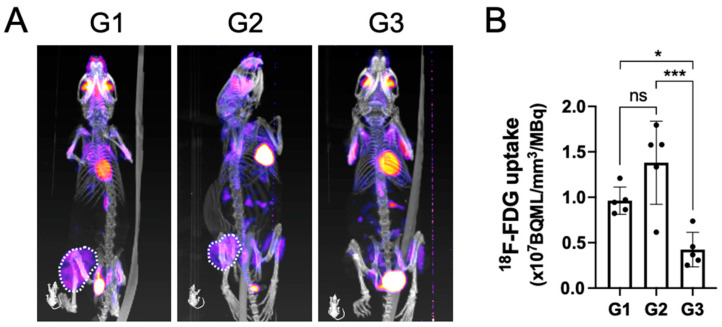
The metabolically active tumor volume after being treated by surgical resection followed by local implantation of nHA + DOX composites detected by PET-CT. (**A**) shows the representative PET-CT images indicating ^18^F-FDG uptake within the tumor. (**B**) shows the quantification of ^18^F-FDG uptake in the tumor, which indicates the metabolic activity within the tumor. * indicates *p* < 0.05, *** indicates *p* < 0.001, ns = non-significant.

PET-CT analysis at the terminal time point indicated that the tumors in both group 1 and group 2 were metabolically active, as seen by a large area of positive PET signal (dashed white line) in the proximal tibia (Figure 3A).

Contrary to groups G1 and G2, the tracer uptake in G3 had an obvious reduction. Upon quantifying the volume of the metabolically active tumor, G3 demonstrated a significantly lower tracer uptake when compared with both G1 and G2, which corroborated well with the findings on gross tumor pathology observed macroscopically (Figure 3B).

### 3.3. The Evaluation of Tumor-Induced Bone Destruction by Micro-CT 

**Figure 4:**
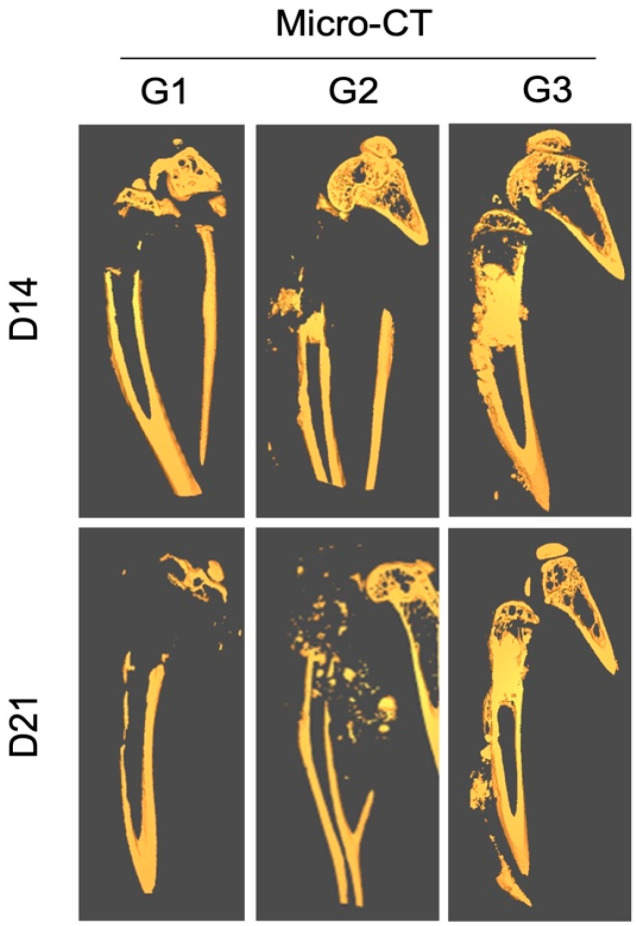
Effect of local treatment with nHA + DOX on preventing bone destruction in an aggressive osteosarcoma in the proximal tibia of nude mice. The representative CT images from day 14 (**top**) and 21 (**bottom**) are presented. Notice that the majority of the proximal tibia and the knee joint is resorbed in G1 and G2, while the proximal tibia is protected in G3.

The in vivo micro-CT analysis performed at 2 weeks post-treatment showed signs of joint destruction in groups G1 and G2, in which a majority of the proximal tibia was resorbed, characteristic of an aggressive osteolytic osteosarcoma (Figure 4).

The epiphyseal bone and cartilage in the proximal tibia could still be observed. In contrast, the tumor area in G3 was filled with radio-dense nHA material. The shape of the bone was normal and the proximal tibia was intact. 

Ex vivo micro-CT performed at 3 weeks showed a similar pattern of bone destruction in groups G1 and G2 as was observed at 2 weeks. However, the bone destruction had advanced into the diaphyseal bone and the cortical bone showed signs of thinning distally. Even at the terminal time-point the proximal tibia and the knee joint in G3 were preserved well and no significant leakage of the implanted HA particles could be observed outside of the medullary cavity. 

### 3.4. Evaluation of Bone Destruction by Histological Analysis

**Figure 5:**
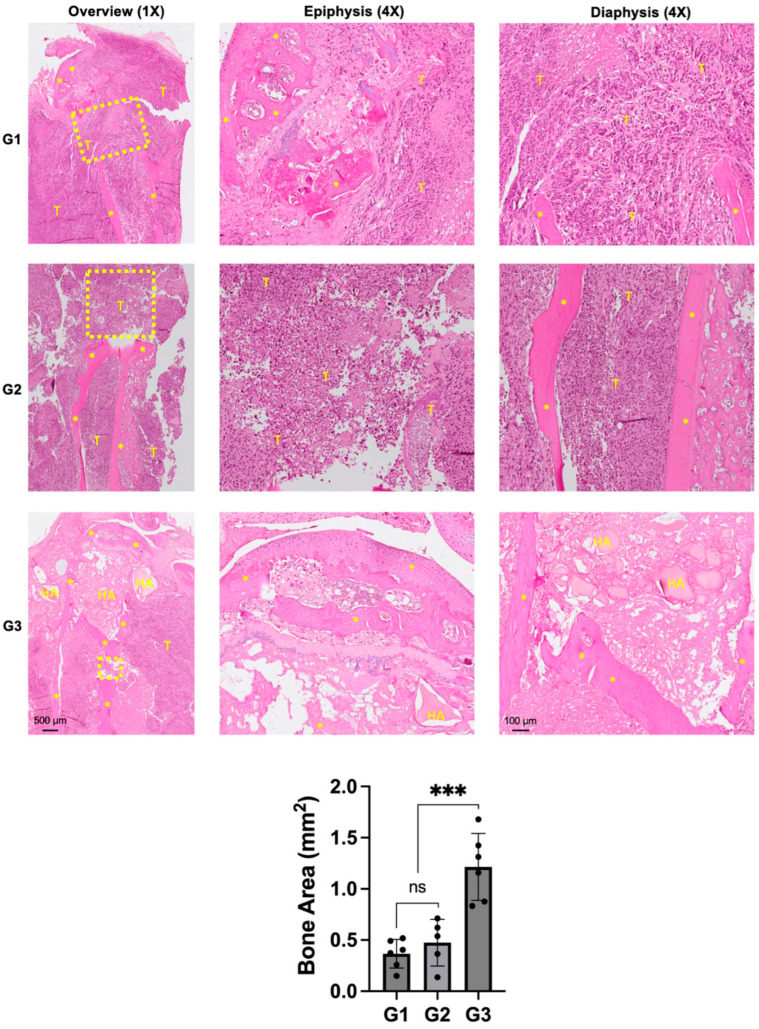
Effect of nHA + DOX on preventing bone destruction in an aggressive osteosarcoma of the proximal tibia. Representative H&E-stained images showing the microscopic structure of the proximal tibia in different groups. * indicates bone, HA represents nano-HA particles and T represents tumor tissue. Dashed yellow box indicates the area of bone destruction in the proximal tibia. Images on the left provide an overview at 1× magnification while images in the middle and right are captured at 4× magnification. The graph at the bottom provides a quantification of the bone area measured in different treatment groups. *** indicates *p* < 0.001 and ns = non-significant.

Histologically, both groups G1 and G2 showed a massive tumor infiltrate in the proximal tibia (Figure 5). 

Scattered chunks of resorbed bone could also be seen in the proximal tibia. The epiphyseal area in G1 and G2 were not completely intact and indicated signs of tumor infiltration into the knee joint and posteriorly into the soft tissue. G3 demonstrated an intact epiphyseal and metaphyseal component. The drill-hole was filled with nHA particles and the bone around the implanted particles appeared to be healthy and did not show any signs of necrosis. Although the bone appeared to be protected from the tumor-induced destruction, soft tissue posterior to the bone was infiltrated by cancer cells. Quantification of the remaining bone area in the proximal tibia corroborated with the osteolysis observed in the micro-CT images, with G3 exhibiting significantly larger bone area compared with both groups G1 and G2. 

### 3.5. Effect of nHA + DOX Delivery on Vital Organs

**Figure 6:**
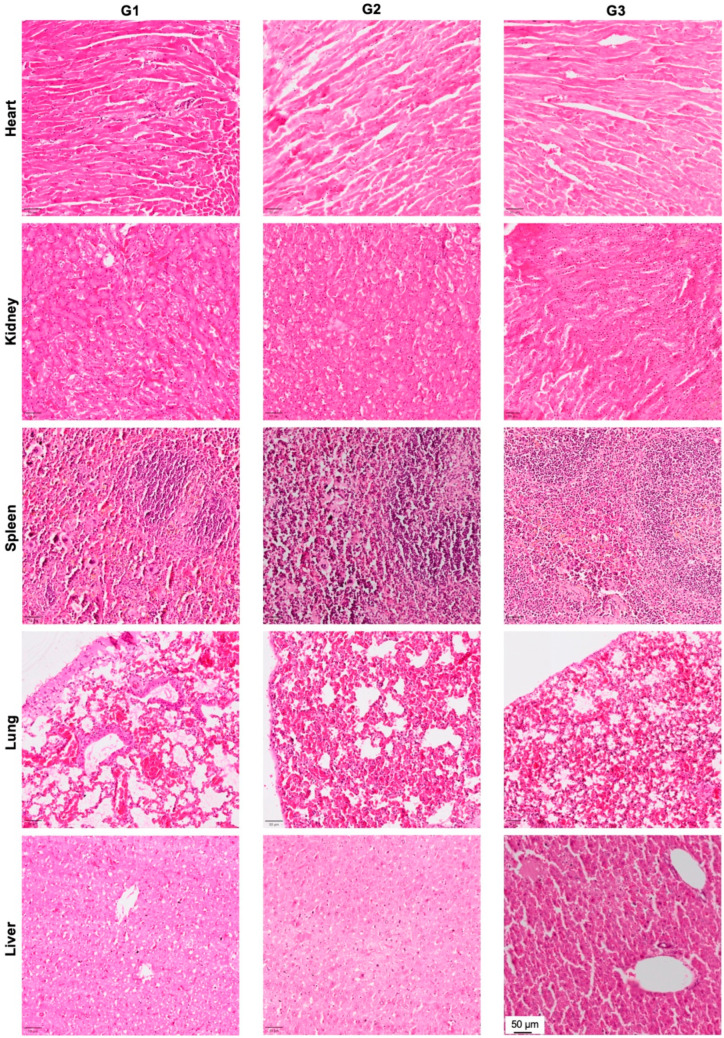
Effect of local DOX delivery mediated by nHA particles on vital organs. The heart, kidney, spleen, lung and liver were stained with H&E and the pictures are captured at 10× magnification to highlight the key histological features of the stained organs.

In order to evaluate the effect of local DOX delivery in G3 on vital organs, we performed H&E staining of the heart, kidney, spleen, lung and liver (Figure 6). No signs of organ toxicity could be observed in any of the vital organs.

## 4. Discussion

Osteosarcoma is still one of the most difficult solid tumors to treat, particularly when the cancer is in its advanced stages. In this study, we found that even after macroscopic surgical resection of the tumor, the remaining tumor cells could repopulate and thereby cause significant damage to the bone. Most importantly, we demonstrated that locally implanted nHA + DOX composite acted as an efficient local drug delivery depot, significantly hampering the growth of the tumor during the study period. Based on the results from this study, it could be envisaged that a HA-based local DOX delivery system could be an efficient complementary treatment of osteosarcoma. The developed treatment could be applied in the following ways: (1) During the pre-operative chemotherapy phase by a direct intra-tumoral injection; (2) As a filler in the resected void to prevent local recurrence; (3) In situations where a tumor is located in an inoperable anatomical location. 

In recent years, various biological targets or antibodies have been explored as treatments for multiple cancers, and in particular for osteosarcoma [14,15,21]. However, very few of the experimental therapies have translated into the clinic [22,23]. One of the potential reasons for this discrepancy could be the animal model used in such studies, including the subcutaneous xenograft model, which relies on tumor growth by injecting cancer cells under the skin. The subcutaneous xenograft model allows the growth of human cancer cells in nude mice to form a mature tumor, but the subcutaneous placement does not mimic the bone microenvironment nor the cross-talk involved in the pathogenesis of osteosarcoma [24]. Therefore, in this study, we inoculated human osteosarcoma cells intra-osseously into the proximal tibia to better mimic the clinical situation. Cell inoculation was performed by using a collagen scaffold as a fast-degrading cell carrier to enable cell retention in the implanted site, which consequently reduces biological variation. Furthermore, another important reason to create an orthotopic model was the lack of metastatic lesions previously observed in the subcutaneous xenograft. Although we could successfully demonstrate tumor development subcutaneously after using 143B cells, lack of pulmonary metastasis made us shift to the orthotopic tumor model, which has previously shown to induce metastases in vital organs [25]. Using the scaffold-based cancer cell inoculation, we successfully elucidated the development of an orthotopic human osteosarcoma xenograft in the mouse tibia, without affecting the surrounding soft tissues. 

We have previously shown that DOX interacts electrostatically with HA, and HA nanoparticles loaded with DOX may lead to better tumor eradication by targeting mitochondria [18]. However, the efficacy of a nHA + DOX delivery system has not been tested in an orthotopic tumor model. To mimic the post-surgical clinical scenario, we resected the tumor using a handheld drill. In the control group (G1), we saw that the tumor reoccurred even after the macroscopic resection, which indeed validated the clinical resemblance of the animal model. In the pristine nHA group (G2), nHA particles were filled into the defect after the tumor resection. This was done similarly for the DOX-functionalized nHA in G3. The application of the particles in the resected void was uneventful despite the small size of the mouse bones. The results showed that nHA + DOX composites lead not only to better tumor eradication but also to reduced bone destruction. The current treatment regimen for osteosarcoma in patients comprises of three cytostatics and therefore the addition of other cytostatics delivered via the HA nanoparticles could eventually provide an even better tumor-killing effect [26,27]. 

Apart from the primary goal of tumor eradication in osteosarcoma, the repair of the bone defect after the tumor resection is also a very important research topic [28]. The first step is to protect the healthy surrounding bone from being destroyed by the tumor cells. This has hardly been explored before. In this study, we showed that surgical resection plus implantation of nHA + DOX composites preserved more healthy bone compared to the control groups. This local DOX delivery approach could therefore protect the bone from resorption while the HA, which is a natural component of bone, could act as an osteoconductive substrate for new bone formation. In order to further boost the osteoconductive properties of HA, other biological and chemical molecules known to accelerate bone regeneration without affecting the tumor should be explored in order to simultaneously eradicate the tumor and replace the tumor by healthy bone [29].

Systemic chemotherapy involves multiple treatment cycles and DOX is known to be cardiotoxic at cumulative doses above 550 mg/m^2^. Off-target side effects after multiple chemotherapy cycles is common as only <10% of the drug reaches the tumor. Apart from cardiotoxicity, DOX can also lead to a toxic response in other vital organs, including the heart, liver and kidney [30]. By means of local and controlled delivery of DOX via HA nanoparticles, it is envisaged that the systemic levels of free DOX can be kept to a minimum. Another important aspect to consider when using nanoparticle-mediated delivery of drugs is the risk associated with nanoparticle migration and off-target organ accumulation [31]. To evaluate the microstructure of vital organs, we did sections for vital organs, including the liver, kidney, lungs and spleen. From the histology, no obvious pathological changes were detected and furthermore, no HA particles could be observed in the histological sections of the vital organs. Our group recently used radioactively (^14^C)-coupled HA particles implanted in the proximal tibia of rats to track the in-vivo migration of HA nanoparticles into the systemic circulation and vital organs. Using scintillation counting and micro-CT imaging, we were able to show that >99% of the particles were retained locally in the bone and no off-target effect could be noticed [32]. These findings, combined with the findings of the current study, strongly suggest that nano-HA particle-based delivery of DOX in a bone void could be a safe treatment modality with limited risk of HA-induced toxicity. 

There are several limitations in this study. First of all, we could not quantify the bone destruction from the micro-CT data due to the difficulty in separating bone and dense HA particles. Secondly, we did not study the systemic toxic effects of the local DOX delivery approach and neither could we elucidate the pharmacokinetics of DOX in the systemic circulation as well as in the tumor. This was predominantly because of the size limitations associated with the use of mouse models, which limited the amount of HA particles that could be implanted intratumorally. Further studies in large animal models are required to elucidate both the pharmacokinetics as well as the analysis of molecular and histopathological markers of systemic toxicity. One might also argue that the lack of systemic DOX treatment group in the study design would have highlighted the role of local DOX delivery even further. However, we have in our previous two studies repeatedly demonstrated that the use of systemic DOX does not perform better than the negative control group (no treatment group) in this aggressive human osteosarcoma. Finally, we have only used one specific local DOX concentration in our study and further pre-clinical studies will be required to elucidate the most optimal DOX dose required to elucidate an optimal tumor response. 

## 5. Conclusions

In this study, we report that the local and controlled delivery of DOX in an orthotopic osteosarcoma using a nanosized HA carrier significantly halts tumor growth as well as bone destruction in an experimental mouse model. Further pharmacokinetics studies in large animal models as well as material rheology, intra-tumoral injectability and drug biodistribution must be evaluated before a translation of the developed approach into clinical studies will be possible.

## Data Availability

The original contributions presented in the study are included in the article. Any further inquiries can be directed to the corresponding author.
